# Coniferyl Aldehyde Attenuates Radiation Enteropathy by Inhibiting Cell Death and Promoting Endothelial Cell Function

**DOI:** 10.1371/journal.pone.0128552

**Published:** 2015-06-01

**Authors:** Ye-Ji Jeong, Myung Gu Jung, Yeonghoon Son, Jun-Ho Jang, Yoon-Jin Lee, Sung-Ho Kim, Young-Gyo Ko, Yun-Sil Lee, Hae-June Lee

**Affiliations:** 1 Division of Radiation Effects, Korea Institute of Radiological and Medical Sciences, Seoul, Korea; 2 College of Veterinary Medicine, Chonnam National University, Gwangju, Korea; 3 Department of Life Sciences, Korea University, Seoul, Korea; 4 College of Pharmacy & Division of Life & Pharmaceutical Sciences, Ewha Womans University, Seoul, Korea; National Cancer Institute, UNITED STATES

## Abstract

Radiation enteropathy is a common complication in cancer patients. The aim of this study was to investigate whether radiation-induced intestinal injury could be alleviated by coniferyl aldehyde (CA), an HSF1-inducing agent that increases cellular HSP70 expression. We systemically administered CA to mice with radiation enteropathy following abdominal irradiation (IR) to demonstrate the protective effects of CA against radiation-induced gastrointestinal injury. CA clearly alleviated acute radiation-induced intestinal damage, as reflected by the histopathological data and it also attenuated sub-acute enteritis. CA prevented intestinal crypt cell death and protected the microvasculature in the lamina propria during the acute and sub-acute phases of damage. CA induced HSF1 and HSP70 expression in both intestinal epithelial cells and endothelial cells *in vitro*. Additionally, CA protected against not only the apoptotic cell death of both endothelial and epithelial cells but also the loss of endothelial cell function following IR, indicating that CA has beneficial effects on the intestine. Our results provide novel insight into the effects of CA and suggest its role as a therapeutic candidate for radiation-induced enteropathy due to its ability to promote rapid re-proliferation of the intestinal epithelium by the synergic effects of the inhibition of cell death and the promotion of endothelial cell function.

## Introduction

Radiation therapy is used in at least 50% of patients with cancer and plays a crucial role in 25% of cancer cures [1]. Despite advances in radiation treatment techniques, radiation toxicity to normal tissue remains the biggest obstacle. The intestine is one of the most susceptible organs to radiation toxicity [2]. During radiation therapy of tumors in the abdominal cavity and pelvis, intestinal regions are inevitably included in the treatment field and represent important healthy tissues at risk (reviewed in [1]). Most patients treated with abdominal radiation suffer from some degree of acute radiation enteropathy, with symptoms such as nausea, abdominal pain, diarrhea, and fatigue, and delayed damage such as fibrosis is observed in ~10% of patients. Thus, alleviating radiation-induced enteropathy is important to improve cancer treatment and patient quality of life.

Radiation-induced enteropathy is a complex pathophysiological process. Acute intestinal injury is the result of direct cell death in the rapidly proliferating crypt epithelium and of inflammation, resulting in insufficient replacement of the villus epithelium and sequential breakdown of the mucosal barrier. Epithelial injury and diarrhea significantly contribute to early radiation-induced morbidity and mortality, which are integrally linked to endothelial apoptosis and vascular dysfunction, resulting in the transfer of intravascular fluids to the gut lumen [3, 4]. The pathogenesis of delayed intestinal injury involves mucosal atrophy, intestinal wall fibrosis, and microvascular sclerosis, all of which are irreversible effects [1].

Endothelial injury due to irradiation (IR) is a key event in the initiation of damage to normal tissues [5]. Extensive apoptosis of microvascular endothelial cells of the lamina propria leads to the development of a primary lesion initiated by intestinal radiation damage [3]. In addition, microvascular endothelial cell apoptosis leads to the loss or dysfunction of crypt stem cell clonogens [4]. Experimental data suggest that the prevention of endothelial cell damage by growth factors such as vascular endothelial cell growth factor (VEGF), basic fibroblast growth factor (bFGF), and an angiopoietin-1 variant inhibit crypt cell damage, organ failure, and death in radiation-induced gastrointestinal syndrome [6, 7].

Coniferyl aldehyde (CA) is a phenolic compound extracted from plants such as *Cinnamomum cassia* [8], *Senra incana* [9], *Ficus foveolata* [10], and *Eucommia ulmoides* [11–13], all of which are well-known ingredients of Asian traditional medicines that are used to treat hypertension, to reinforce muscles and bones, and to recover damaged liver and kidney function. CA possesses five-fold higher anti-inflammatory activity than that of aspirin [9] as well as potent anti-platelet aggregation activity [8]. Our previous study revealed a new function of CA as a potent inducer of heat shock factor 1 (HSF1), which upregulates heat shock proteins (HSPs), including HSP27 and HSP70 [14]. HSPs protect cells from various stimuli, including oxidative stress, heat, and radiation [15, 16]. In this study, we further investigated the radioprotective effects of CA in intestinal damage. CA significantly alleviated radiation-induced enteropathy by increasing endothelial cell survival and eventually allowing the intestine to recover.

## Materials and Methods

### Ethics statement

All mouse procedures in this study were approved by the Institutional Animal Care and Use Committee of the Korea Institute of Radiological and Medical Sciences (IACUC permit number: KIRAMS2013-028).

### Reagent

CA was purchased from Sigma-Aldrich (382051, St. Louis, MO).

### Animals and experimental procedures

**Fig 1:**
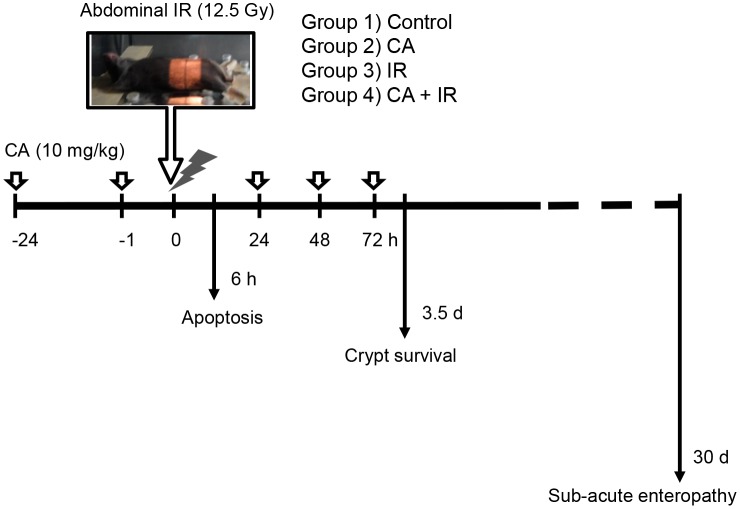
Experimental scheme of the protective effects of CA on abdominal IR-induced radiation enteropathy. Abdominal IR was performed using X-Rad320, as shown in the photo. CA (10 mg/kg per dose) was intraperitoneally administered to C3H mice at 24 h and 1 h before and 24, 48 and 72 h after IR. The mice were euthanized at 6 h after IR for apoptosis detection and at 3.5 days after IR for crypt survival assays (*n* = 6/group). To assess sub-acute radiation enteropathy, 6 mice from the control and CA-only groups and 15 mice from the IR and CA and IR combination groups were sacrificed at 30 days after abdominal IR.

C3H mice were obtained from Doo-Yeol Biotech Co. Ltd. (Korea) at 7 weeks of age (average body weight, 18.2 ± 2.1 g). The mice were maintained for 1 week prior to the experiments and randomly assigned to the following groups: 1) control (n = 18); 2) CA (n = 18); 3) IR (n = 27); and 4) CA + IR (n = 27). The animals were housed in a specific pathogen-free facility and were fed a normal diet and autoclaved water *ad libitum*. CA (10 mg/kg, intraperitoneal injection; *i*.*p*.) was administered at 24 and 1 h before and 24, 48 and 72 h after IR. The control and IR group were injected with same volume of vehicle, which is 5% dimethyl sulfoxide (DMSO) in saline. Each mouse assigned to the radiation treatment group was exposed to radiation under anesthesia (30 mg/kg Zoletil and 10 mg/kg Rompun) using an X-Rad320 (Precision X-Ray, East Haven, CT; filter: 2 mm AI; 42 cm, 260 kV/s, 10 mA, 2.0 Gy/min). The radiation field size for localized abdominal exposure was 30 × 50 mm. After abdominal IR, the mice were euthanized at the time point indicated in Fig 1, and histopathological analysis was performed.

### Histopathological analysis

A 10-cm segment of the jejunal intestine was collected, fixed in formalin, and embedded in paraffin wax. To analyze morphological changes, all samples were sectioned and reoriented in successive slices to identify those containing the longest villi [17]. Two sections from 4 different parts of the jejunum from each animal were prepared for histological examination. Immunohistochemistry was conducted using a Vectastain Elite ABC Kit (Vector Laboratories Inc., CA, USA), and apoptotic cell death was assessed using a Dead End Fluorometric TUNEL System (G3250, Promega, WI, USA), following the manufacturers’ protocols. For antigen retrieval, the sections were boiled in citrate buffer (pH 6.0). The sections were then incubated overnight at 4°C with an anti-PECAM1 antibody (1:100, sc-1506, Santa Cruz Biotechnology, TX, USA) and an anti-Ki-67 antibody (1:200, DRM004, Acris Antibodies, Germany) and washed with PBS containing 0.05% Triton X-100. The sections then were incubated with the corresponding secondary antibody for 30 min and counterstained with hematoxylin or DAPI.

To detect apoptotic cells, TUNEL-positive signals were counted in forty longitudinal crypt sections showing a large proportion of cells in the crypt base and lumen and in at least 17 cells along the crypt column and in the lamina propria of the 10 longest villi per slice using a microscope [18]. To identify PECAM1-positive cells, PECAM1-positive areas were measured in the 10 longest villi and their lamina propria using the image analysis program of a confocal microscope.

To analyze the jejunal crypt and the morphological changes in sub-acute enteropathy, the numbers of crypts and villi in jejunal cross-sections were counted in 10 slices from 4 different parts of each mouse. Additionally, the heights of the 10 longest villi in a single slice were measured. PECAM1 expression was measured and quantified in the lamina propria of the 10 longest villi in 4 single slices for each animal.

### Cells

Human umbilical vein endothelial cells (HUVECs) were purchased from Promocell and maintained in Endothelial Growth Medium-2 (EGM-2; Promocell GmbH, Heidelberg, Germany) in 0.1% gelatin-coated dishes. The IEC6 rat intestinal epithelial cell line and SNU449 human hepatocellular carcinoma cell line were obtained from Korean Cell Line Bank (Seoul, Korea). The AGS human gastric cancer cell line and RKO human colon carcinoma cell line were obtained from ATCC (USA). The IEC6 and cancer cell lines were cultured in Dulbecco’s Minimum Essential Medium (WELGENE, Daegu, Korea) supplemented with 10% fetal bovine serum and antibiotics and incubated in 5% CO_2_ at 37°C. Scrambled siRNA (si-con), HSF1 siRNA (si-HSF1) and HSP70 siRNA (si-HSP70) were purchased from Santa Cruz Biotechnology (TX, USA) and transfected using Lipofectamine 2000 (Invitrogen, USA) in Opti-MEM for 24 h before the CA and IR treatments were performed.

### Western blot analysis

Mouse intestinal tissues were physically minced in Pro-prep solution (17081, Intron Inc., Korea), and then proteins were extracted from the lysates. Total proteins were extracted from cells using RIPA lysis buffer. Western blot analysis was performed using the following antibodies: HSP70 (1:1000, C92F3A-5, Enzo Life Sciences, NY), HSF1 (1:1000, sc-17757, Santa Cruz Biotechnology), eNOS (1:1000, sc-634, Santa Cruz Biotechnology), cleaved caspase-3 (1:1000, #9961, Cell Signaling, MA), cleaved PARP (1:1000, #9542 Cell Signaling), and β-actin (1:3000, Sigma, MO). The blots were incubated with these antibodies overnight at 4°C.

### Detection of cell death

A total of 2 x 10^6^ cells were seeded in a 100-mm culture dish, incubated overnight, and treated with CA 3 h before IR. At 72 h after IR, suspended cells were stained using an Annexin V/PI Staining Kit (K101-100, Biovision, CA), following the manufacturer’s protocol. Dead and living cells were detected by flow cytometry.

### Tube formation assay

Matrigel (354230, Corning, MA) was mixed with EGM-2 medium at a 1:1 ratio and used to coat 48-well plates at room temperature. HUVECs were treated with 5 μM CA before IR with 10 Gy for 3 h. Each well was seeded with 4.5×10^4^ cells and incubated at 37°C. Tubule formation by the cells was counted at 16 h after IR.

### Colony formation assay

Viable caner cells were plated into each 60-mm culture dish and allowed to grow following the CA (2.5 μM) and IR (0–6 Gy) treatments at 37°C in a humidified 5% CO_2_ incubator. After 7–10 days, the dishes were stained with 0.4% crystal violet, and colonies (> 50 cells) were counted. The surviving fraction was calculated as the mean number of colonies/cells inoculated × the plating efficiency [19].

### Tumor growth rate analysis

Mouse colon cancer CT26 (purchased from ATCC) cells (1 x 10^6^ in 100 μL HBSS) were injected into the subcutaneous regions of the flanks of 6-week-old male BALB/C mice (Doo-Yeol Biotech). When tumors reached 100 mm^3^ in size, the mice were randomly assigned to 6 groups, as follows: 1) control; 2) CA; 3) 8 Gy IR; 4) CA + 8 Gy IR; 5) 12.5 Gy IR; and 6) CA + 12.5 Gy IR (n = 5). CA (10 mg/kg, *i*.*p*.) was administered to mice 5 times before and after IR according to the same schedule used for radiation enteropathy presented in Fig 1. The control and IR groups were injected with same volume of vehicle (5% DMSO in saline). The volume of each tumor was measured 3 times per week for 21 days.

### Statistics

All values are presented as the mean ± standard error of the mean (SEM). The data were analyzed by analysis of variance (ANOVA) and a Bonferroni post hoc test for multiple comparisons. *P*-values < 0.05 were considered to indicate statistical significance.

## Results

### CA protects against acute radiation-induced intestinal injury

**Fig 2:**
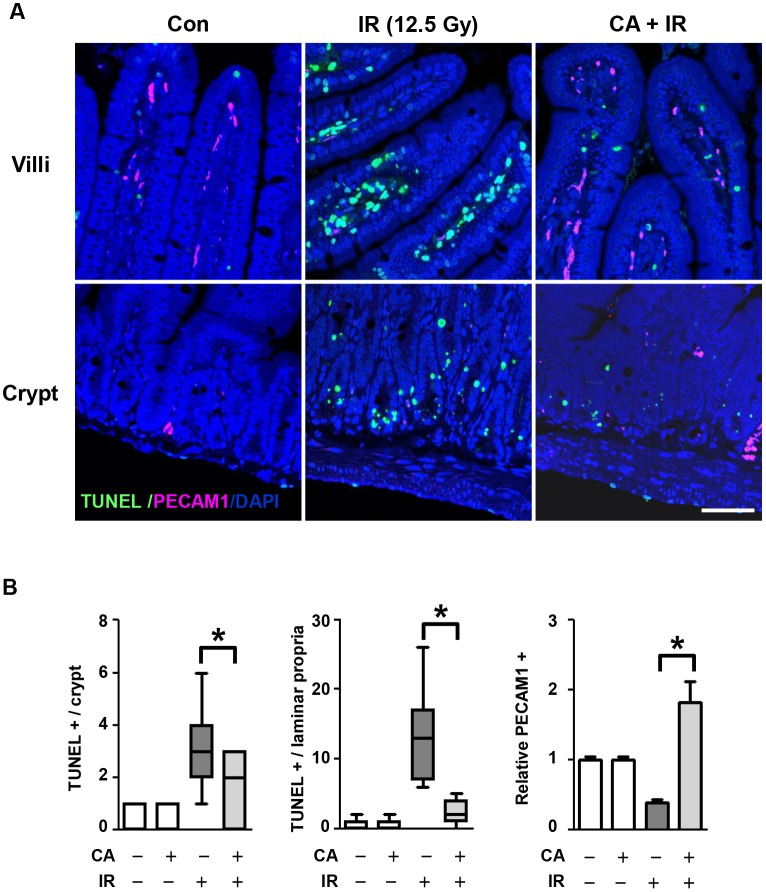
CA treatment inhibits apoptotic epithelial and endothelial cell death after IR. **A**. Apoptosis in the jejunum at 6 h after 12.5 Gy abdominal IR. Apoptotic FITC-TUNEL (green) and endothelial rhodamine-PECAM1 (red) are shown counterstained with DAPI (blue). Scale bar = 30 μm. **B**. The distribution of apoptotic cells in crypts and lamina propria and PECAM1 in a villus unit of the jejunum after 12.5 Gy IR. The values represent the mean ± SEM (*p < 0.05, *n* = 6).

To investigate the effects of CA on the intestine, we reduced radiation-induced bone marrow injury using localized abdominal IR. We exposed the abdomens of mice to 12.5 Gy IR under anesthesia using an Xrad-320, which is designed to localize IR in experimental animals (Fig 1). After the local abdominal IR, we measured apoptotic cell death in the intestine to investigate the direct effects of CA on cell death during IR. We quantified apoptotic cell death in the crypts and lamina propria by TUNEL assay. Apoptosis occurred widely throughout the crypts and lamina propria at 6 h after 12.5 Gy abdominal IR (Fig 2A). The mean rate of apoptotic cell death in the control mice was 0.27 ± 0.14 in the crypts and 0.55 ± 0.21 in the lamina propria, which was not different from that of apoptotic cell death in the CA group (0.27 ± 0.12 in the crypts and 0.64 ± 0.20 in the lamina propria). The rate of IR-induced apoptotic cell death was 5.90 ± 0.34 in the crypts and 13.05 ± 1.47 in the lamina propria. However, CA treatment in the IR group significantly inhibited the incidence of TUNEL-positive cells by 31% (1.80 ± 0.31, *p* < 0.05) in the crypts and by 18% (2.33 ± 0.43) in the lamina propria. Interestingly, PECAM1 expression, a pan-marker of endothelial cells, was significantly increased following CA+ IR treatment versus IR treatment (4.61-fold, *p* < 0.01), suggesting that CA protected not only proliferating crypt cells, including intestinal stem cells, but also endothelial cells.

**Fig 3:**
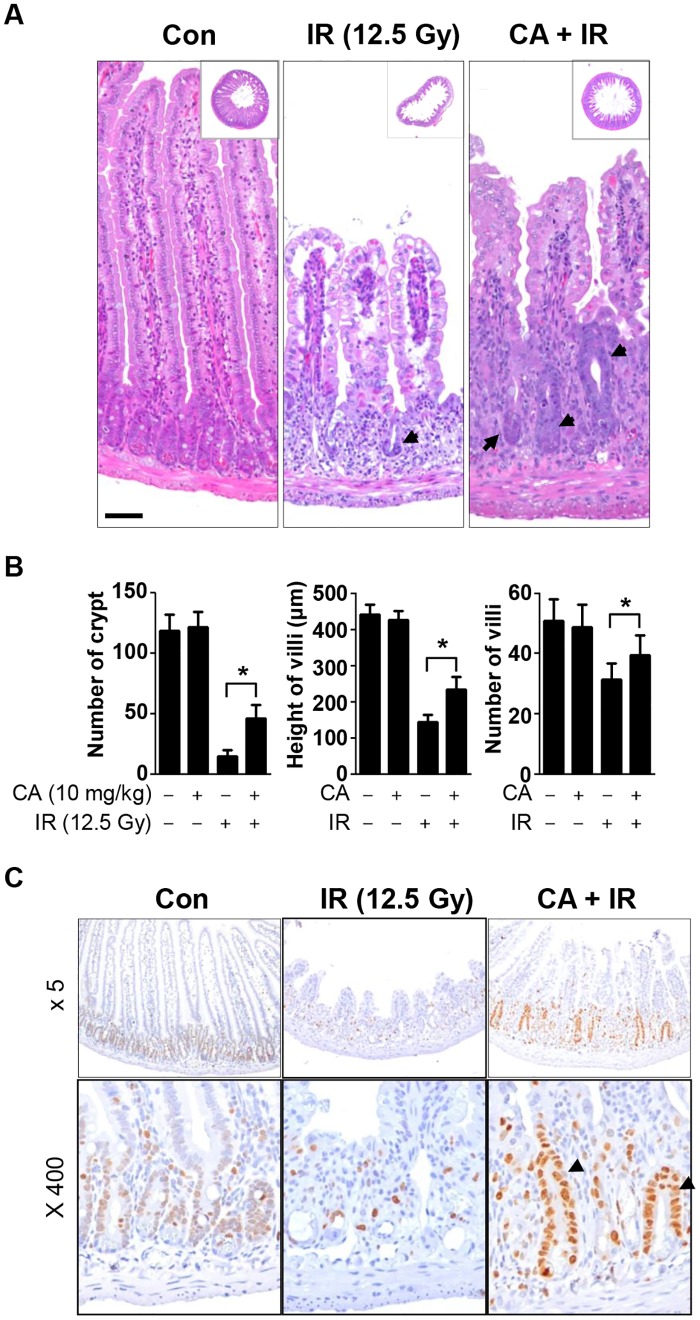
CA treatment rescues jejunal crypt survival after high-dose abdominal IR. **A**. Hematoxylin and eosin (H&E)-stained jejunal sections harvested from vehicle- or CA-treated mice at 3.5 days after 12.5 Gy abdominal IR. The inserted squares depict whole cross sections of the jejunum. Original magnification, 5x. The arrows indicate crypts that survived following IR. Scale bar = 50 μm. **B**. Quantitative analysis of morphological changes in the jejunum following CA and IR. Data are presented as the mean ± SEM (*p < 0.05, *n* = 6). **C**. Immunohistochemical analysis of Ki-67, a proliferation marker, in a section of the jejunum at 3.5 days after IR. Ki-67 was evaluated using diaminobenzidine (DAB, brown stain) and hematoxylin counterstaining (blue). The arrows indicate crypts, representing intestinal stem cell proliferation.

We then measured the effects of CA on crypt survival in jejunum samples collected 3.5 days after abdominal IR at 12.5 Gy. Because intestinal stem cells and proliferative crypt cells are positioned in the crypt, we measured crypt survival to assess the repopulation activity of the injured intestine. After 12.5 Gy IR, remarkable jejunal crypt loss, villi shortening, and vacuole and erythrocyte loss in the villus epithelium and lamina propria were observed (Fig 3A). CA treatment alone did not induce any apparent morphological changes in the intestine compared with the control. CA treatment of the irradiated mice significantly rescued the crypt numbers, villi heights, and villi numbers compared with the IR group by the following percentages: 26% (0.13 ± 0.04 vs. 0.39 ± 0.10, *p* < 0.01), 20% (0.33 ± 0.05 vs. 0.53 ± 0.08, *p* < 0.01), and 16% (0.62 ± 0.10 vs. 0.78 ± 0.13, *p* < 0.01), respectively (Fig 3B). To assess proliferative activity, we performed immunohistochemistry for the proliferation marker Ki-67. Weak Ki-67staining was observed in most crypt cells in the control group. The intestines of the IR group showed few Ki-67-stained cells, whereas those of CA + IR group showed strong Ki-67 staining of cells in the crypt and some cells in the lamina propria (Fig 3C).

### CA attenuates sub-acute radiation enteropathy

**Fig 4:**
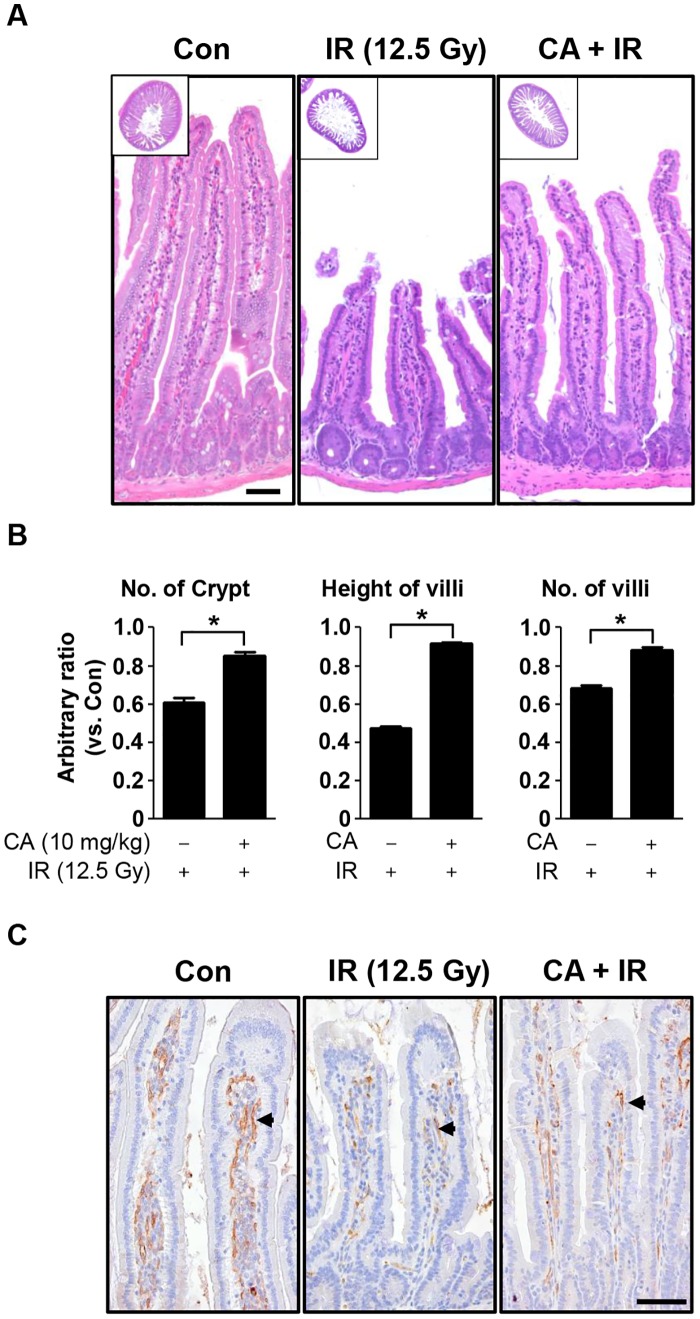
CA treatment ameliorates radiation-induced enteritis. **A**. A H&E-stained section from mice treated with CA at 30 days after 12.5 Gy abdominal IR. Scale bar = 50 μm. Inserted squares show whole cross sections of the jejunum. Original magnification, 5x. **B**. Quantitative analysis of morphological changes in the jejunum following IR. Numbers of crypts, villi heights, and numbers of villi are shown. The data are presented as the mean ± SEM (*p < 0.05, *n* = 6). **C**. Immunohistochemical analysis of PECAM1 in jejunal sections. PECAM1 was detected using DAB (brown) and hematoxylin counterstaining (blue). Scale bar = 25 μm. The arrows indicate PECAM1-positive cells.

To investigate the long-term effect of CA on radiation enteropathy, we evaluated the survival of mice for 30 days after IR localized to the intestine. The loss of body weight began on the first day after IR; the animals reached their minimum body weights after 7 days, after which they slowly recovered until 21 days after IR (S1 Fig). The animals exposed to localized abdominal IR had low mortality (7% for the IR treatment and 0% for the CA + IR treatment, n = 15). The mice in IR group showed severe diarrhea, which began at 3 days and persisted for 14 days, after which melena, indicating upper intestinal bleeding, was observed until the end of the experimental period (30 days). However, the mice in CA + IR group exhibited mild diarrhea from 3 to 10 days, weak melena for 1 week, and then a return to ‘normal’ conditions before 21 days. CA treatment also ameliorated the IR-induced loss of body weight (S1A Fig). On day 30, we sacrificed the animals and harvested the intestines for histological analyses. In terms of gross pathology, intestines of the IR group displayed thinner walls and smaller diameters compared with those of the CA-treated IR group (S1B Fig). Histopathological analyses revealed apparently normal structures of the epithelial cells of the villi, with single columnar epithelia and no hemorrhage or vacuolization at 30 days after IR compared with those at 3.5 days after IR. However, the intestines of IR group still showed reduced numbers of crypts, markedly shortened villi, and reduced numbers of villi, which were reduced by 25%, 44%, and 20%, respectively (*p* < 0.01; Fig 4A and 4B) compared with the CA-treated group. Additionally, increased collagen deposition was observed in the lamina propria of the IR group compared with the CA + IR group (S2 Fig). PECAM1 expression in the lamina propria was significantly increased in the CA+IR group compared with the IR group (Fig 4C).

### HSF1 and HSP70 upregulation by CA protects against radiation-induced cell death

**Fig 5:**
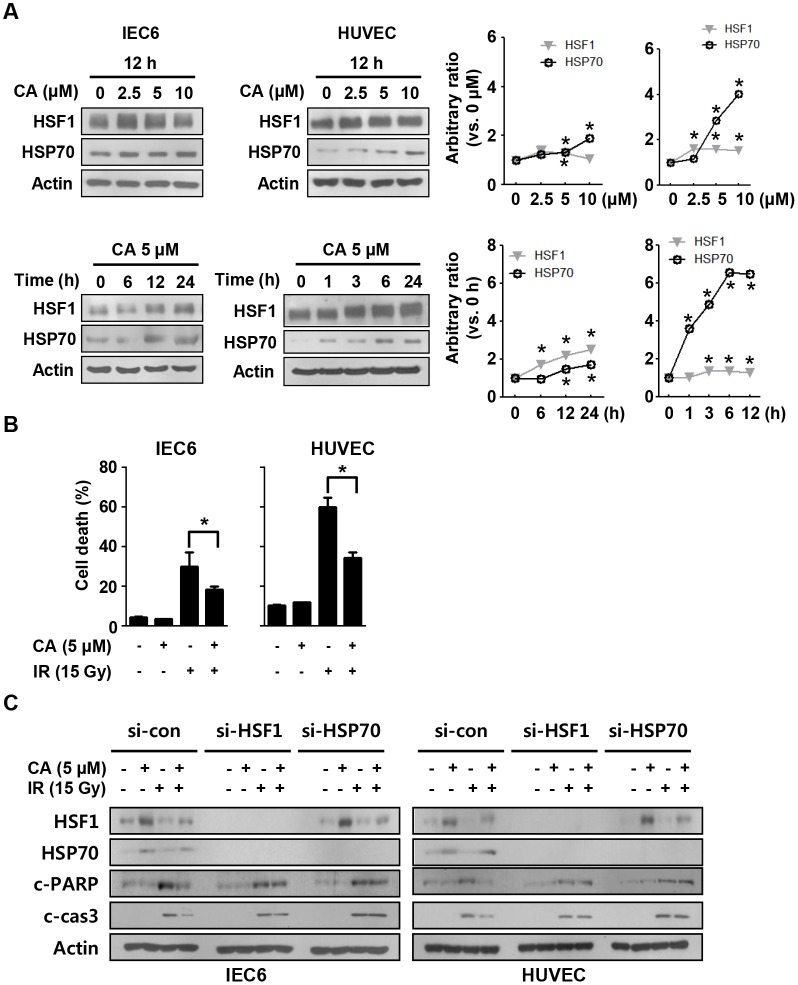
CA confers protection against radiation-induced cell death by inducing HSF1 and HSP70 expression. **A**. HSF1 and HSP70 expression in IEC6 cells and HUVECs was detected by Western blotting after treatment with 0–10 μM of CA at the time points indicated. **B**. Apoptotic cell death was measured by Annexin V/PI assay and flow cytometry at 48 h after 15 Gy IR. **C**. IEC6 cells and HUVECs were transfected with si-con, si-HSF1 or siHSP70 and treated with 5 μM of CA and 15 Gy of IR. Western blotting was conducted to detect cleaved caspase-3 and cleaved PARP at 48 h after 15 Gy IR in IEC6 cells and HUVECs. Actin was used as a loading control. Three different experiments were performed.

Because systemic CA treatment effectively protected the intestine according to the histological analyses, we sought to confirm the protective effects of CA *in vitro*. We treated IEC6 cells, which are rat intestinal epithelial cells with stem cell properties, and HUVECs with various doses of CA at different time points. CA treatment upregulated HSF1 and HSP70 in both IEC6 and HUVECs at doses higher than 2.5 μM within 12 h after treatment (Fig 5A). A single dose of CA (10 mg/kg, *i*.*p*.) in mice induced HSF1 and HSP70 expression in the intestine within 24 h (S3 Fig). Apoptotic cell death measurements using PI and Annexin V staining and FACS following 5 μM CA treatment and 15 Gy IR revealed that CA decreased the rate of apoptosis by 8% in IEC6 cells and by 20% in HUVECs compared with the IR group (Fig 5B). In agreement with the FACS data, CA treatment decreased cleaved caspase-3 and cleaved PARP levels following IR exposure in both IEC6 cells and HUVECs. However, these protective effects were blocked following si-HSF1 or si-HSP70 treatment (Fig 5C).

### CA promotes endothelial cell function mediated by HSF1

**Fig 6:**
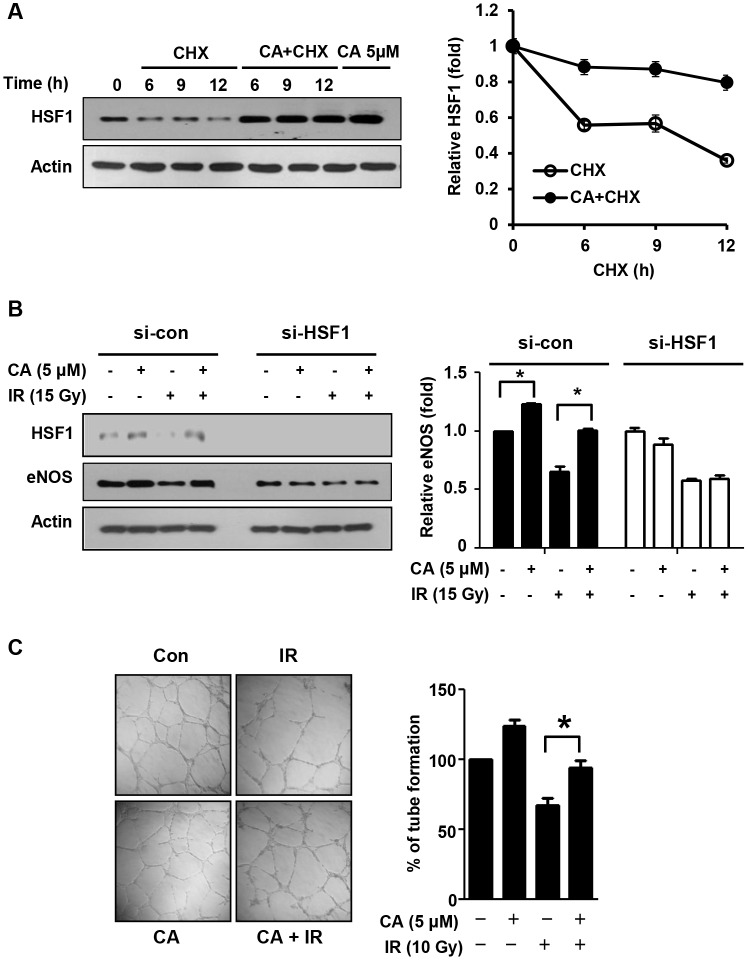
CA promotes endothelial cell function via HSF1. **A**. HUVECs were incubated in the presence of cycloheximide (CHX, 10 μg/ml) with or without 5 μM of CA and were analyzed by Western blotting (*p < 0.05 vs. control cell). **B**. HUVECs were transfected with si-con and si-HSF1, and the expression of HSF1 and eNOS was analyzed after 48 h of CA treatment and IR. **C**. A tube formation assay in Matrigel was performed using HUVECs. HUVECs were treated with 5 μM CA for 1 h before exposure to 10 Gy IR. The cells were detached from a 100-mm dish, and 4.5×10^4^ cells were seeded on Matrigel in a 96-well plate at 24 h after IR. After 16 h of incubation, the tubes on the Matrigel were counted, and the results are depicted as a graph (**p < 0.01, *n* = 3).

Because CA effectively protected endothelial cells in the mouse intestine, we examined its effect on endothelial cells. Following treatment with cycloheximide (CHX; 10 μg/ml), control cells exhibited rapid HSF1 protein degradation, whereas CA-treated cells exhibited prolonged HSF1 stability (Fig 6A). Additionally, CA treatment increased eNOS in HUVECs and inhibited loss of eNOS by IR; these effects were abolished with si-HSF1 treatment (Fig 6B). Next, to assess the effects of CA on the angiogenic activity of endothelial cells, we conducted tube formation assays using HUVECs. Tubes were disrupted in the IR-only group at 16 h after 10 Gy IR, whereas CA treatment preserved most of the tubes (Fig 6C). Additionally, HUVECs treated with CA alone displayed slightly increased tube formation activity compared with control cells.

### CA does not contribute to cancer cell protection against IR

**Fig 7:**
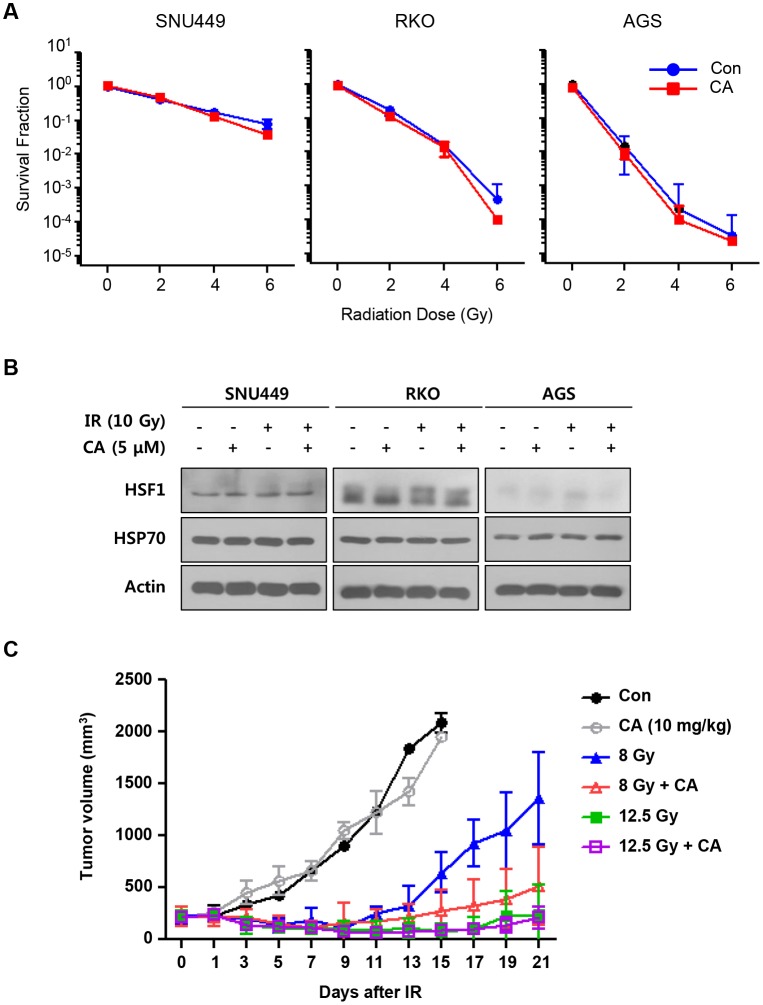
CA treatment did not protect tumors against IR. **A**. Colony formation assays were performed with human cancer cell lines. Cells were seeded and cultured 7 to 10 days following CA treatment (0 and 2.5μM) and IR (0–6 Gy). **B**. HUVECs were transfected with si-con or si-HSF1 and treated with 5 μM of CA and 15 Gy of IR. Western blotting was conducted to detect HSF1 and eNOS at 48 h after IR. Actin was used as a loading control. Three different experiments were performed. **C**. The effect of CA on tumor growth was measured in CT26 allografts in BALB/c mice. Five CA doses (10 mg/kg, *i*.*p*.) were administered to tumor-bearing mice before and after 8 or 12.5 Gy IR. Tumor volumes were measured three times per week for 21 days. The data are presented as the mean ± SEM (*p < 0.05, *n* = 5).

To consider whether CA has protective effects against cancer, we assessed cancer cell proliferation and colony formation assays following CA and IR treatments in three human cancer cell lines. Neither CA alone nor the combined treatment with CA and IR altered cell proliferation or the surviving fraction of cancer cells (S4A Fig and Fig 7A). To evaluate HSF1 and HSP70 induction in three cancer cell lines following CA and/or IR treatment, we confirmed the expression levels of these proteins using Western blotting. CA alone or combined with IR did not induce HSF1 or HSP70 expression in the cancer cell lines (Fig 7B and S4B Fig). Finally, we treated these cells with CA (five intraperitoneal injections of 10 mg/kg) at the same dose that showed protective effects on the normal intestines of CT26 mouse colorectal cancer cell line-bearing mice. Administering 12.5 Gy abdominal IR to CT26-bearing tumors suppressed tumor growth during 3 weeks, and 8 Gy IR prevented tumor growth until 9 days after IR and then tumors regrew. Combination of 8 Gy IR and CA treatment showed more preventive effect on tumor regrowth compared to 8 Gy IR alone however there was no statistical significance. Thus, CA did not effect on tumor growth with or without IR (Fig 7C).

## Discussion

Due to concerns regarding radiation exposure after an accident or from terrorist activity, research interest has increased for developing medical countermeasures against the radiation-induced gastrointestinal (GI) tract injury [20, 21]. Although radiotherapy is effective and is widely used, normal tissue toxicity remains the greatest problem, particularly for the medical use of radiation. The GI and hematopoietic systems are highly sensitive to radiation, and GI syndrome and myelosuppression are major causes of death following radiation exposure [22, 23]. Thus, most protectors or mitigators of radiation damage that have been developed target intestinal and bone marrow injury.

We investigated the effects of CA on radiation enteropathy with a localized abdominal IR model because CA possesses anti-inflammatory activity, which is effective for treating ulcerative colitis and intestinal inflammatory disorders [24]. A survival test using 8 Gy total-body IR, which is a relatively high dose, revealed that CA modestly prolonged survival time compared with the control in C3H mice (S5 Fig). Restricting radiation exposure to the abdomen attenuates radiation-induced bone marrow damage and likely sensitizes mice to lethality from GI syndrome [25]. We also chose 12.5 Gy for the localized abdominal IR dose because endothelial cell death occurs at doses exceeding 10 Gy, whereas those exceeding 14 Gy may affect survival [26, 27]. In most cases of pelvic or abdominal radiotherapy, patients suffer acute enteropathy but generally have a very low mortality rate and few severe chronic impediments (< 5% of patients). We observed only one death in the IR group during the 30 days of the experiment; however, following 12.5 Gy abdominal IR, all animals showed serious intestinal damage, with severe crypt injury and apoptotic cell death during the acute or sub-acute phases of enteropathy as well as enteritis. Whole-abdominal IR over 10 Gy is designed for developing possible radioprotective agents to protect from GI tract damage [17, 28]. The incidence and severity of delayed intestinal radiation toxicity depends on the radiation dose, the volume of the bowel being irradiated, the fractionation schedule, the concomitant chemotherapy, and the co-morbidities and other patient factors [29]. Therefore, our model is more effective for observing longer–term effects, including the side effects of RT during the acute- and sub-acute phases, and may also be used for developing countermeasures for accidental radiation exposure.

To evaluate the acute effects of CA on intestinal radiation injury, we measured apoptotic cell death at 6 h after IR because the early wave of apoptosis caused by radiation occurs at 3–6 h after IR [30]. CA significantly inhibited apoptotic cell death in both crypts and villi, indicating that CA prevented direct DNA damage by IR (Fig 2). This direct protective effect of CA on apoptotic cell death contributed to crypt survival at 3.5 days after IR, resulting in reduced tissue injury and enhanced proliferative activity, as shown in Fig 3, and in subsequent recovery from sub-acute radiation enteropathy of the remaining stem cells, which are necessary to reconstitute the crypts and villi (Fig 4). Therefore, CA treatment preserves epithelial and endothelial cells during the acute phase following IR, allowing for potential re-population of the intestinal components, including crypt stem cells, and the microvasculature, which also has effects during the delayed phase following IR.

In this study, we found that CA significantly inhibited apoptotic cell death, which was blocked in the absence of HSF1 or HSP70 (Fig 5). The heat shock response is an adaptive response to various proteotoxic stresses, and the major heat shock transcription factor 1 (HSF1) facilitates cell proliferation and survival in eukaryotes [31]. Our data are consistent with other reports that HSP70 overexpression confers protection against DNA damage by ultraviolet [32] and ionizing radiation [33] via DNA repair and that HSF1 improves cell survival following IR. Moreover, human HSF1 improves vascular endothelial cell function via PAI-1 and ET-1 suppression and eNOS and thrombomodulin upregulation [34]. CA also increases HSF1 protein stability and expression by activating HSP70 transcription and mediates the increased HSF1 stability by phosphorylating HSF1 at Ser326 [35]. In this study, we confirmed that CA increased HSF1 stability in HUVECs and altered eNOS mediated HSF1 expression, which are key molecules controlling endothelial cell function. Further, CA treatment increased angiogenesis and protected this process from the effects of IR *in vitro* (Fig 7). We found that the levels of PECAM1, an endothelial marker, and the numbers of rescued crypts were close to their normal values following CA treatment, whereas the IR group showed endothelial cell loss throughout the intestine. In the same tissue, we found that CA treatment rescued the heights of the villi more significantly than the other parameters such as the numbers of crypts and villi at 30 days after IR. Thus, endothelial cell protection by CA may contribute to its synergic effects on preventing or reducing radiation-induced intestinal injury.

To assess the protective effects of CA on cancer, we tested its effects on human cancer cell lines and CT26-bearing mice. CA did not alter cancer cell proliferation or clonogenic activity *in vitro* and did not affect tumor growth or regrowth *in vivo* (Fig 7). The exact mechanism by which CA regulated HSF1 and HSP70 in cancer cells remains unknown, but CA does not induce HSF1 or HSP70 in cancer cell lines (S4 Fig). Because CA mediated HSF1 and HSP70 upregulation, we speculate that it did not affect cancer cell proliferation or growth. Therefore, CA is a therapeutic candidate and a mitigator of radiation-induced normal tissue injury and does not cause adverse effects in cancer patients.

In this study, we demonstrated that CA significantly alleviated radiation enteropathy and mediated HSF1 upregulation. The induction of HSF1 by CA and its transcriptional regulation of HSP70 protected epithelial and endothelial cell death and endothelial cell function from IR. Our data support the further investigation of CA as a potential therapeutic agent for radiation enteropathy.

## Supporting Information

S1 Fig Gross intestinal lesions following IR and CA. **A**. Changes in the body weights of mice following vehicle and CA treatments at 30 days after IR. **B**. Photo of intestinal tissue harvested at 30 days after 12.5 Gy local abdominal IR.
(TIF)

S2 Fig CA attenuates radiation-induced enteropathy. Masson’s trichrome staining was performed to detect collagen deposition in jejuna harvested from CA-treated mice at 30 days after 12.5 Gy abdominal IR. The arrows and stars indicate a fibrotic region of the lamina propria.
(TIF)

S3 Fig The induction of HSF1 and HSP70 expression by CA in mouse intestinal tissue. Western blotting for HSF1 and HSP70 in intestinal tissue lysates harvested at 24 h after a single *i*.*p* dose of 10 mg/kg CA.
(TIF)

S4 Fig The effect of CA on human hepatocellular carcinoma, gastric and colorectal cancer cell lines. **A**. MTT assay results for the SNU449, AGS and RKO cell lines. The cell lines were treated with 5 μM CA for 12 h before IR. At 48 h after IR, the MTT reagent was added to the medium and incubated for 2 h at 37°C, and absorbance measurements were obtained using a spectrophotometer. **B**. Western blotting for HSF1 and HSP70 in cancer cell lines. CA treatment was applied at five concentrations to human gastric and colon cancer cell lines for 12 h, and then Western blotting was performed.
(TIF)

S5 Fig Survival curves for CA treatment in mice following 8 Gy total body irradiation. C3H mice were exposed to 8 Gy total body irradiation (TBI) following CA treatment compared with IR group. TBI was performed using an X-Rad320 (Precision X-Ray, East Haven, CT; filter: 2 mm AI; 42 cm, 260 kV/s, 10 mA, 2.0 Gy/min). The radiation field size was 100 × 100 mm. The animals received CA (10 mg/kg) at 24 and 1 h before and 24, 48, and 72 h after 8 Gy IR. The IR group was injected with same volume of vehicle (5% DMSO in saline). Following 8 Gy TBI, death of the animals occurred beginning at 5 days after IR, and 100% mortality was reached within 10 days. In the CA+IR group, radiation-induced mortality also began at 5 days after IR but ended at 13 days, and the mean survival time was prolonged by 30 h compared with the IR group (201 ± 15 h in IR group vs. 231 ± 13 h in the CA + IR group, *p* < 0.05).
(TIF)

S1 Methods (DOC)

S1 Checklist (PDF)
