# Supplementary figures and images for: Coniferyl Aldehyde Attenuates Radiation Enteropathy by Inhibiting Cell Death and Promoting Endothelial Cell Function
Source: PLoS One. 2015 Jun 1;10(6):e0128552. doi: 10.1371/journal.pone.0128552 (PMC4452689; doi:10.1371/journal.pone.0128552)

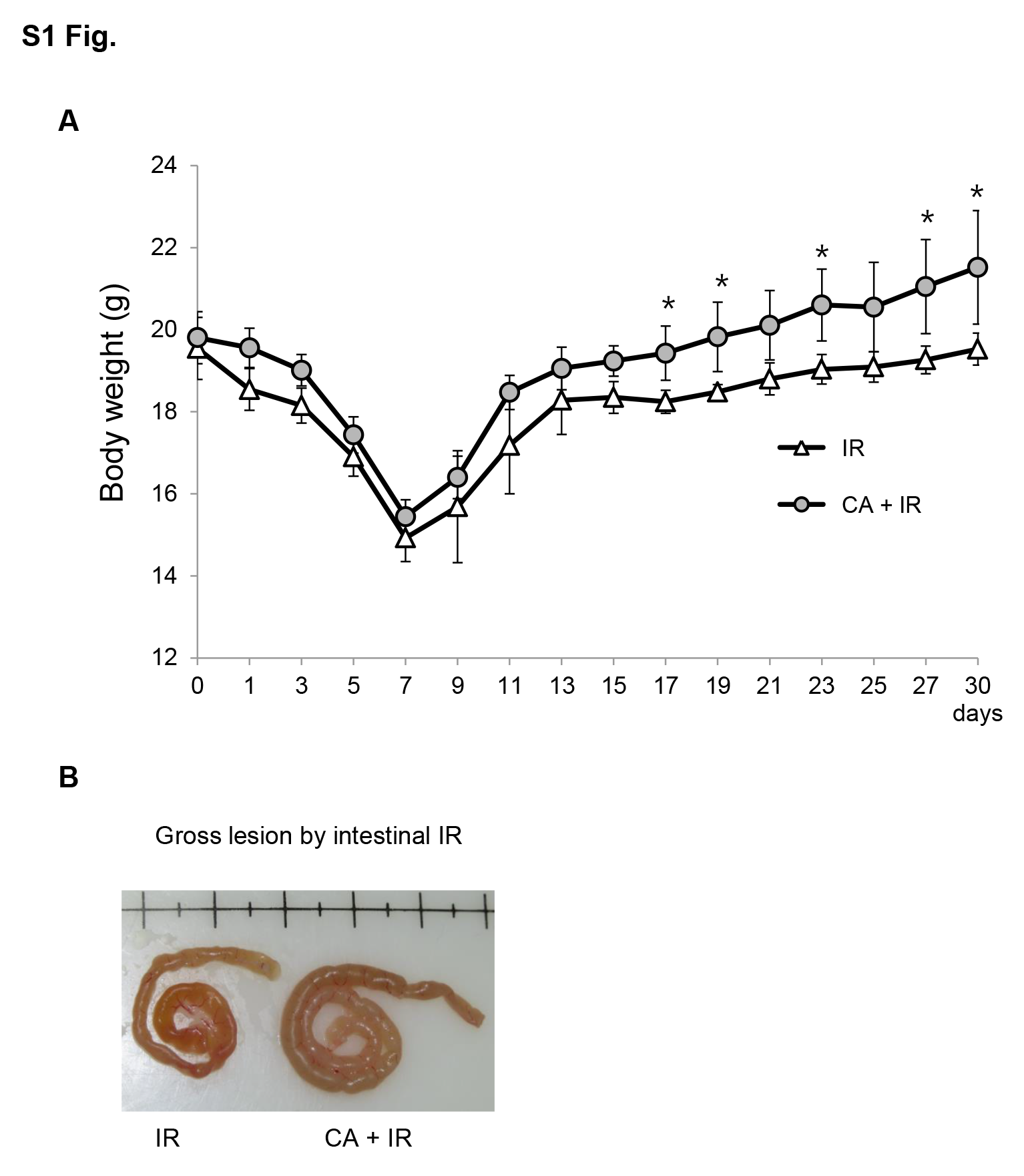

Supplement: S1 Fig — A. Changes in the body weights of mice following vehicle and CA treatments at 30 days after IR. B. Photo of intestinal tissue harvested at 30 days after 12.5 Gy local abdominal IR. (TIF) [file pone.0128552.s001.tif]

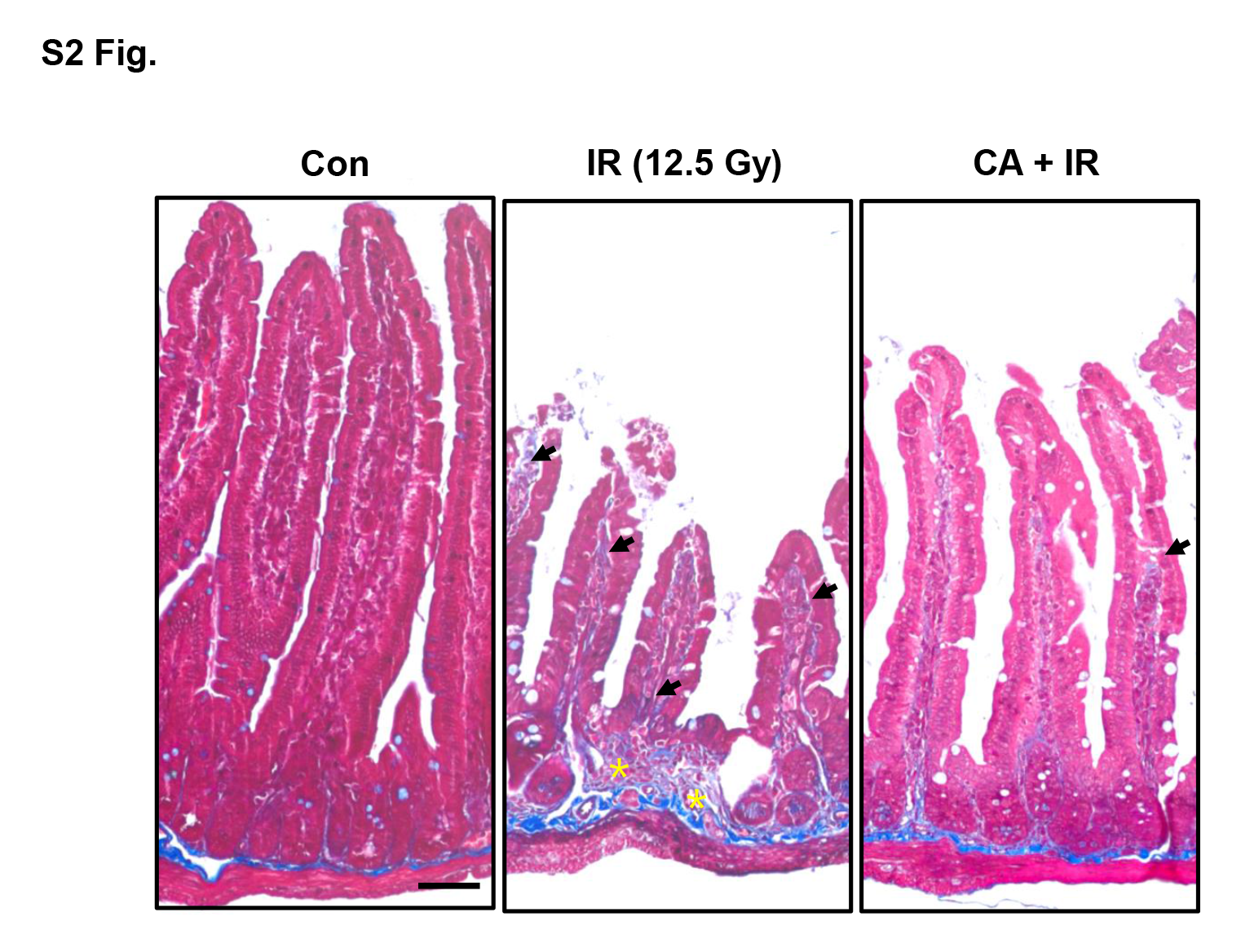

Supplement: S2 Fig — Masson’s trichrome staining was performed to detect collagen deposition in jejuna harvested from CA-treated mice at 30 days after 12.5 Gy abdominal IR. The arrows and stars indicate a fibrotic region of the lamina propria. (TIF) [file pone.0128552.s002.tif]

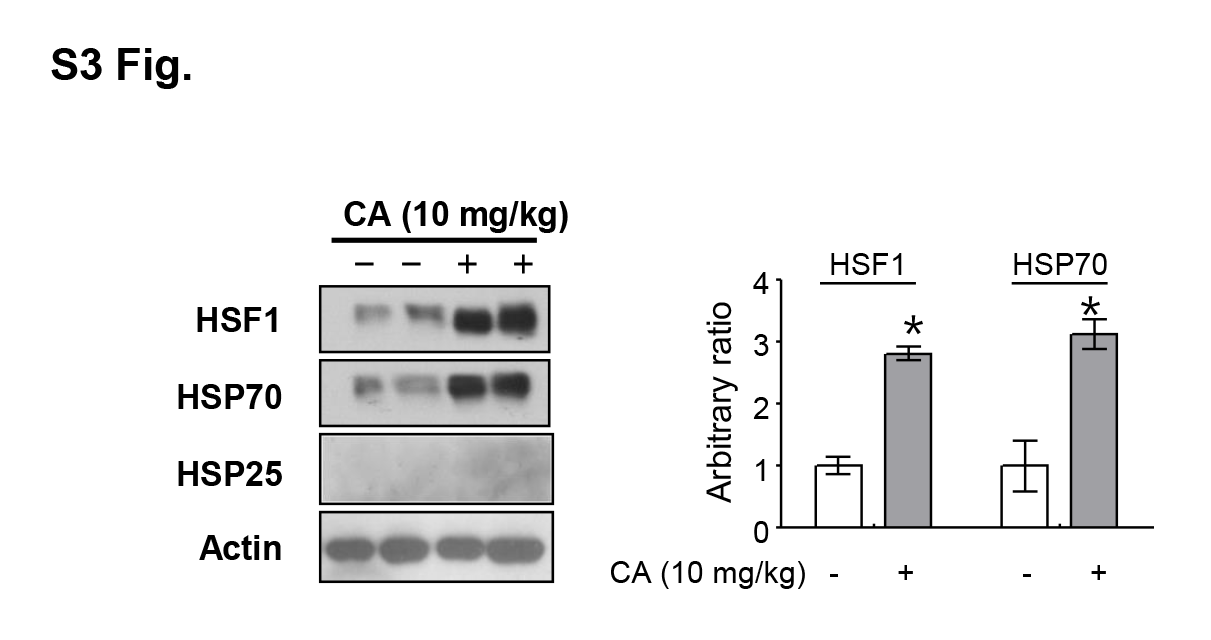

Supplement: S3 Fig — Western blotting for HSF1 and HSP70 in intestinal tissue lysates harvested at 24 h after a single i.p dose of 10 mg/kg CA. (TIF) [file pone.0128552.s003.tif]

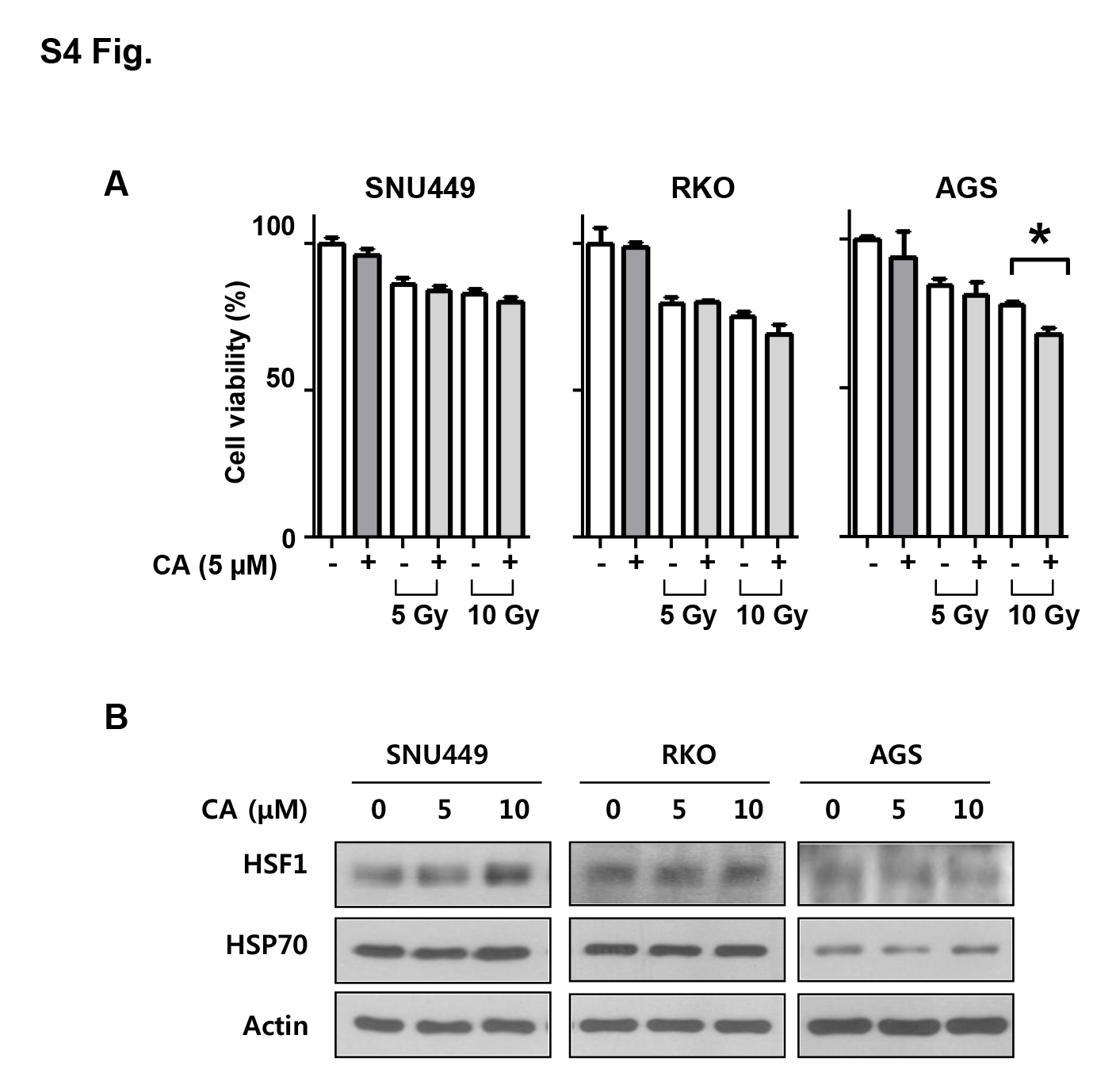

Supplement: S4 Fig — A. MTT assay results for the SNU449, AGS and RKO cell lines. The cell lines were treated with 5 μM CA for 12 h before IR. At 48 h after IR, the MTT reagent was added to the medium and incubated for 2 h at 37°C, and absorbance measurements were obtained using a spectrophotometer. B. Western blotting for HSF1 and HSP70 in cancer cell lines. CA treatment was applied at five concentrations to human gastric and colon cancer cell lines for 12 h, and then Western blotting was performed. (TIF) [file pone.0128552.s004.tif]

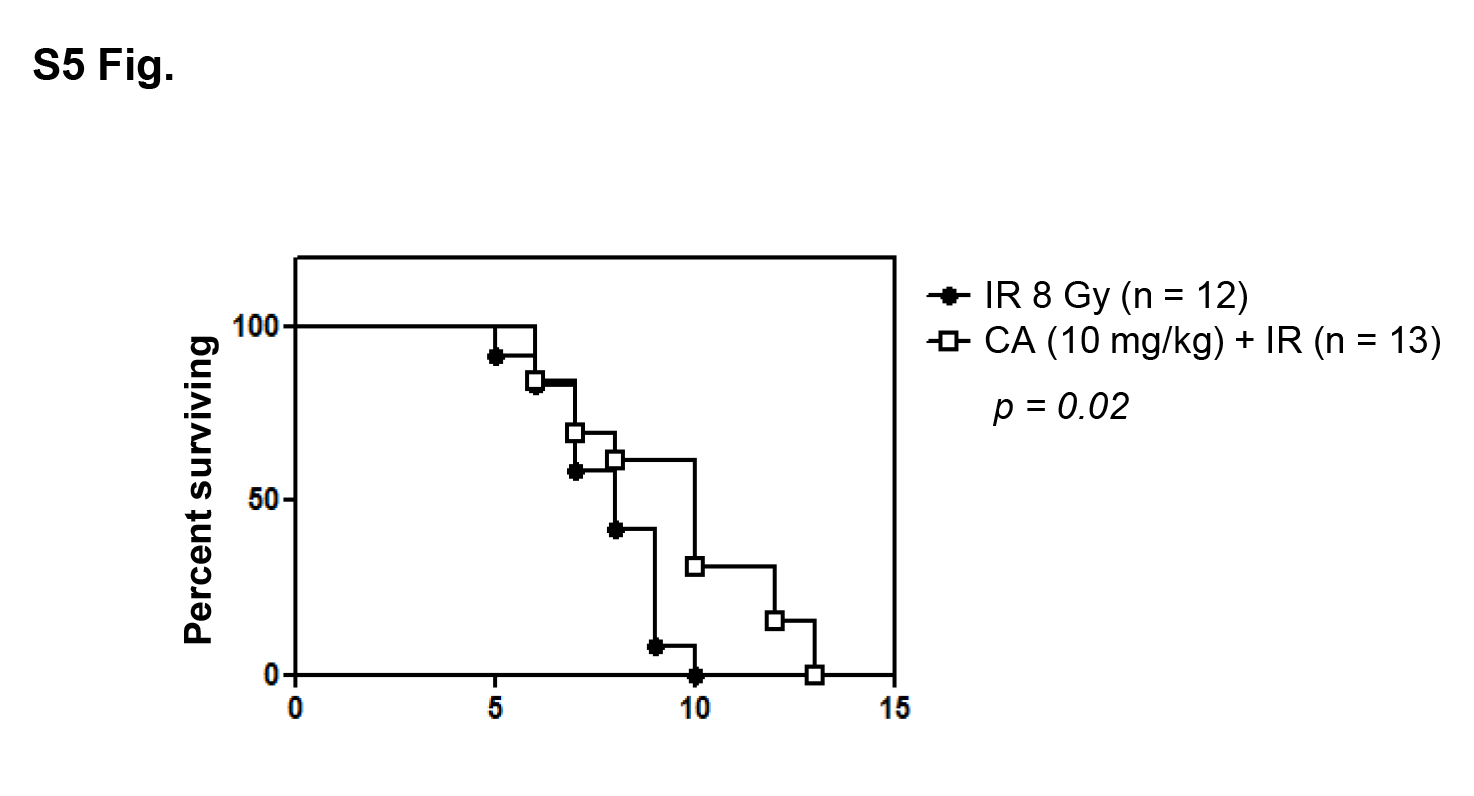

Supplement: S5 Fig — C3H mice were exposed to 8 Gy total body irradiation (TBI) following CA treatment compared with IR group. TBI was performed using an X-Rad320 (Precision X-Ray, East Haven, CT; filter: 2 mm AI; 42 cm, 260 kV/s, 10 mA, 2.0 Gy/min). The radiation field size was 100 × 100 mm. The animals received CA (10 mg/kg) at 24 and 1 h before and 24, 48, and 72 h after 8 Gy IR. The IR group was injected with same volume of vehicle (5% DMSO in saline). Following 8 Gy TBI, death of the animals occurred beginning at 5 days after IR, and 100% mortality was reached within 10 days. In the CA+IR group, radiation-induced mortality also began at 5 days after IR but ended at 13 days, and the mean survival time was prolonged by 30 h compared with the IR group (201 ± 15 h in IR group vs. 231 ± 13 h in the CA + IR group, p < 0.05). (TIF) [file pone.0128552.s005.tif]
