# Supplementary material for: Coniferyl Aldehyde Attenuates Radiation Enteropathy by Inhibiting Cell Death and Promoting Endothelial Cell Function
Source: PLoS One. 2015 Jun 1;10(6):e0128552. doi: 10.1371/journal.pone.0128552 (PMC4452689; doi:10.1371/journal.pone.0128552)
Supplement: S1 Methods — (DOC) [file pone.0128552.s006.doc]

**S1 Methods.**

**Masson’s trichrome staining**

Masson’s trichrome staining was performed as follows: formalin-fixed, paraffin wax-embedded sections were deparaffinized with xylene and rehydrated. After fixing in Bouin’s solution at 56°C for 1 h, the slides were counterstained with hematoxylin and then stained using a trichrome staining kit (HT15-1KT, Sigma-Aldrich), following the manufacturer’s protocol.

**MTT assay**

Cells (1×10^4^/well) were seeded in a 96-well plate, incubated overnight, treated with CA at 3 h before IR, and incubated for another 48 h after IR. MTT (thiazolyl blue tetrazolium bromide, M2128, Sigma-Aldrich, MO) solution was then added to the cells and incubated for 2 h at 37°C, following the manufacturer’s protocol. Optical densities were assessed using a spectrophotometer.

**Survival test**

For the survival test, mice were divided into the following treatment groups: vehicle, CA, 8 Gy total-body IR and 12.5 Gy abdominal IR. The health of the animals was evaluated each day during the experiment according to a protocol that was approved by the Institutional Animal Care and Use Committee of the Korea Institute of Radiological and Medical Sciences. Our experimental protocol permitted one exception, which was to euthanize animals only if they had lost more than 25% of their body weight, as pilot studies have indicated that radiation typically induces the loss of body weight up to 25% until 7 days. Bodyweight was recorded every day after the experiment was initiated. Animals were euthanized before the indicated time points if they showed the following signs of death: 1) inappetence: complete anorexia for 24 h; 2) weakness/inability to obtain food or water: inability or extreme reluctance to stand, persisting for 24 h; 3) moribund state: measured by a lack of a sustained purposeful response to gentle stimuli (an example of a purposeful response is a weak attempt to get up; if the animal is on its side, attempts should be asymmetrical in nature); 4) infection involving any organ system; and 5) signs of severe organ system dysfunction that are non-responsive to treatment or that have a poor prognosis, as determined by a veterinarian.
